# The Effects of the Urban Built Environment on Mental Health: A Cohort Study in a Large Northern Italian City

**DOI:** 10.3390/ijerph121114898

**Published:** 2015-11-20

**Authors:** Giulia Melis, Elena Gelormino, Giulia Marra, Elisa Ferracin, Giuseppe Costa

**Affiliations:** 1 Environmental Heritage and Urban Redevelopment Unit, SiTI—Higher Institute on Territorial Systems for Innovation, via Boggio 61, 10138 Torino, Italy; E-Mail: giulia.marra@polito.it; 2 ASL Torino 5, Local Public Health Agency, piazza S. Pellico 1, 10023 Chieri, Italy; E-Mail: gelormino.elena@aslto5.piemonte.it; 3 SEPI Grugliasco, Epidemiology Service for ASL Torino 3, Local Public Health Agency, via Sabaudia 164, 10095 Grugliasco, Italy; E-Mails: elisa.ferracin@epi.piemonte.it (E.F.); giuseppe.costa@epi.piemonte.it (G.C.)

**Keywords:** built environment, urban structure, Turin Longitudinal Study, health, inequalities, social environment, accessibility, public transport, urban density

## Abstract

Mental health (MH) has a relevant burden on the health of populations. Common MH disorders (anxiety and non-psychotic depression) are well associated to socioeconomic individual and neighborhood characteristics, but little is known about the influence of urban structure. We analyzed among a Turin (Northwest Italy) urban population the association at area level of different urban structure characteristics (density, accessibility by public transport, accessibility to services, green and public spaces) and consumption of antidepressants. Estimates were adjusted by individual socio-demographic variables (education, housing tenure, employment) and contextual social environment (SE) variables (social and physical disorder, crime rates). Data was extracted from the Turin Longitudinal Study (TLS)—a census-based cohort study following up prospectively the mortality and morbidity of the population. As expected, individual characteristics show the strongest association with antidepressant drug consumption, while among built environment (BE) indicators accessibility by public transport and urban density only are associated to MH, being slightly protective factors. Results from this study, in agreement with previous literature, suggest that BE has a stronger effect on MH for people who spend more time in the neighborhood. Therefore, this research suggests that good accessibility to public transport, as well as a dense urban structure (*versus* sprawl), could contribute to reduced risk of depression, especially for women and elderly, by increasing opportunities to move around and have an active social life.

## 1. Introduction

The urban built environment (BE) is one of the potential determinants of health and health inequalities to be considered in the Health in All Policies approach [1]. The health impact of some BE characteristics, such as housing, traffic, environmental pollution and safety, have been widely assessed but little is known about the impact of main structural characteristics of the urban context that are central to urban and local plans, such as land use, building density and distribution of services and facilities.

Mental health (MH) might be considered one of the most responsive health targets of the urban structure. In recent years, studies on BE and MH have primarily focused on two measures: residents’ perceptions of their living environment [2,3] and geographical area variations of the quality of the BE [4,5,6,7,8,9]. The majority of those studies, however, failed to find statistically significant area effects on MH after accounting for individual socioeconomic factors [10].

According to their specific purpose, researchers have applied diverse definitions of BE. What is defined as BE encompasses physical indoor conditions, residential building characteristics and a fuzzy “neighborhood quality” that summarizes a vaguely understood neighborhood disorder. Few studies have clearly identified a pathway between urban BE features (those constituting the public realm [11] rather than indoor conditions) and MH. The association has been suggested and hypothesized as plausible by many of the studies, but it is not always clear to what extent it has been influenced by social pathways (social interaction, social support, *etc.*).

Transport and accessibility research is the only literature that has attempted to isolate the impact of single urban BE features on health. In research carried out by Frank and Savage [12], residential density, land use mix, street connectivity and street design were found to be associated with general health outcomes, but not specifically to MH (link not explored).

Summarizing what is currently known about the influence of single urban features on MH, Duncan [13] described the spatial distribution of BE characteristics in association with depressive symptoms among youth. Dreger [14] revealed that there is a distinct effect on MH resulting from certain physical factors (pollution, poor waste management and road traffic) regardless of psychosocial and socio-demographic components of the neighborhood that are perceived to be positive. For instance, noise emanating from neighbors and lack of access to green open spaces showed an impact on mental well-being [15,16].

Apart from individual socioeconomic factors, some social environment (SE) elements, mainly related to neighborhood disorder and fear of crime, have been discovered to be related to MH [10,17,18,19] and may act as confounders of the BE influence on MH; incivilities against the BE and an absence of safe public areas are also indicated as contributing to MH problems [20].

Depression is frequently reported in the literature as a MH outcome. As a result, antidepressants have been increasingly recommended in recent years for the treatment of both depressive and anxiety disorders [21,22]. A prescription of antidepressants appears to be a useful proxy indicator for all types of depressive symptoms with psychological and/or somatic complaints [23]. Any prescription of antidepressant within a fixed time frame is a better indicator than the total number of prescriptions within that frame: due to variations in access and compliance to antidepressant treatment, it better approximates the mental status of different socioeconomic groups [24,25,26], excluding immigrants, for whom depression is common [27] but undertreated [28].

In conclusion, there is scanty evidence of the impact on and inequalities in MH from the different dimensions of the BE that define the urban structure. This limits the ability of urban planners to take into account the health and health equity impact of their decisions.

The aim of this study is to test the hypothesis that the BE has an effect on MH, irrespective of the roles played by neighborhoods and individual social disadvantage, by looking at variations in antidepressant prescriptions depending on specific dimensions of the BE (urban density, land use mix, green areas, public services, accessibility through public transport).

## 2. Methods

Data was drawn from the Turin Longitudinal Study (TLS), which is based on the city’s historical population registry from 1971 to the present. Demographic information from the registry is individually linked to census variables and routinely registered health events [29,30].

In this study, adult Turin residents were followed up with regarding antidepressant medications prescribed from 1 January 2004 to 31 December 2006, adjusted by individual and contextual socio-demographic circumstances and their residential stability. Men (272, 516) and women (274, 747) aged between 20 and 64 years old, were enrolled and stratified by age ranges (20–34, 35–49, 50–64) in order to account for differences in exposure to neighborhood characteristics by life stage and gender.

Individual socio-demographic conditions were classified according to census data, *i.e.*, educational level (upper secondary education or higher *versus* second stage of basic education or lower (According to ISCED levels (ISCED 0–2 *versus* ISCED > 2)), activity status (active employed *versus* active unemployed and inactive, including housewives, students, retired, other conditions); and population registry data: citizenship (native *versus* migrant) and residential stability (three or more years at the same address *versus* less than three years).

BE and SE characteristics were measured at the neighborhood level (including 79 statistical areas with an average of 10,000 inhabitants each). Some areas (excluded units 13) were excluded from the analysis as they showed extreme values due to their specificity and would invalidate the results. These areas included cemeteries and parks (no residents) and some hilly parts of the city with no services and few inhabitants due to the geographical conformation. We also excluded the wealthy, residential neighborhoods located in the hills of Turin because these areas have geographic characteristics that render them incomparable to the rest of the city in terms of distribution of public facilities and services.

**Figure 1:**
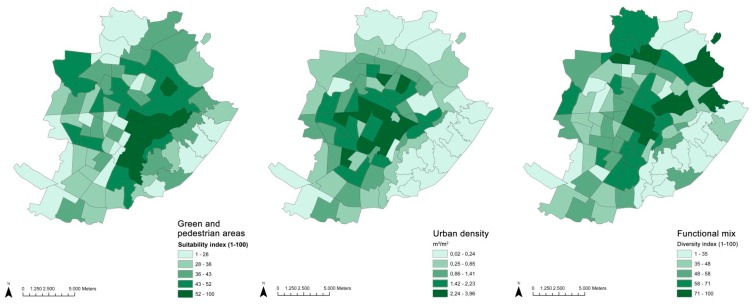
Distribution of BE structural variables.

**Figure 2:**
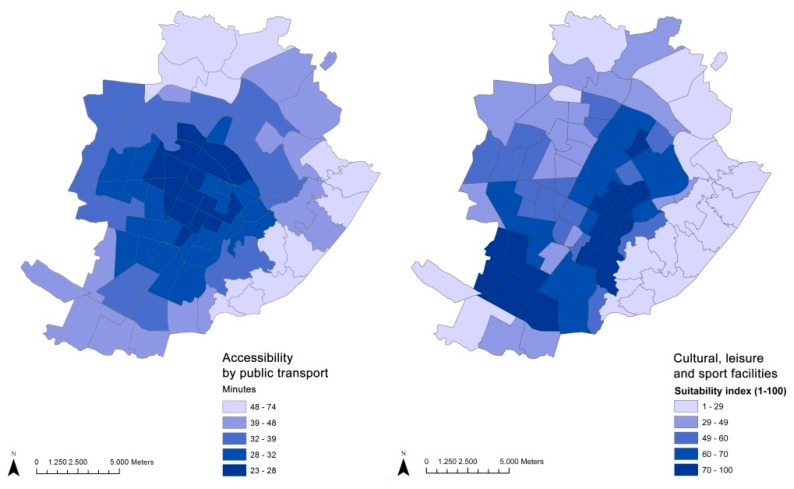
Distribution of BE availability of services.

Data on the BE variables of neighborhoods was collected through the municipality administrative datasets and represent the two main aspects of the city’s profile that are relevant for urban policies, namely the structure of a neighborhood (density, functional mix, and green/pedestrian areas), represented in Figure 1, and the availability of its services (described by cultural and leisure facilities and accessibility by public transport), represented in Figure 2.

The green/pedestrian areas indicator represents the availability of parks, gardens and green facilities (tree-lined shores, forests, gardens, parks, tree-lined squares, river banks, school grounds, various green spaces, excluding green spaces used for sports, which were considered separately) that are within an accessible distance [31] and are combined with an availability of pedestrian areas. Each feature produces an effect on the area which varies according to its size and is maximized on the immediate surrounding until it begins losing its effect at a set distance.

**Figure 3:**
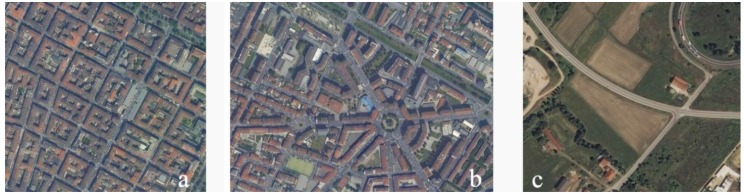
Examples of different urban densities within the city of Turin ((**a**) Highest density = 3.96 m^3^/m^2^; (**b**) Average density = 1.30 m^3^/m^2^; (**c**) Lowest density = 0.02 m^3^/m^2^).

Density was calculated as the ratio of built volume over an area. The built volume, calculated by the multiplication of the sum of the footprint area of all buildings by the eaves height over the total surface of the statistical area varies between 0.02 m^3^/m^2^ for almost non-urban areas (such as the hilly parts of Turin, cemeteries and big parks in the periphery with almost no buildings) to 3.96 m^3^/m^2^ as the maximum value, found in the core of the city and characterized by narrow streets with an average building height of six floors. The mean value for the city is 1.3 m^3^/m^2^, which is an average value common for cities in Italy and indicates an urban setting with a liveable balance of built volume and open space, comprised of a typical composition of blocks, inner courtyards, services and public spaces (Figure 3).

Functional mix—land use mix describing the level of vitality. The land use mix was calculated by applying the Shannon diversity index (1948) [32], which has been utilized in urban planning and land use studies since at least 1994 when it was first applied by Frank and Pivo [33]. We measured the diversity index for each statistical area, looking at five different land uses (residential, productive, tertiary, services and commercial) and normalized values on a scale from 1 to 100.

Cultural and sport facilities is a composite index (normalized between 1–100) which shows the availability at an accessible distance of facilities within the city that are related to free time leisure (public libraries, community centers, cinemas, stadiums and theatres) and sports (gymnasiums, soccer fields, swimming pools, tennis courts) [31].

Accessibility by public transport refers to the amount of time (average in minutes) taken to reach each statistical area from a given one using only public transport. The values range from 23 minutes—the highest score for the best-served zone—to 74 minutes—the lowest value for the worst serviced zone of the city.

**Figure 4:**
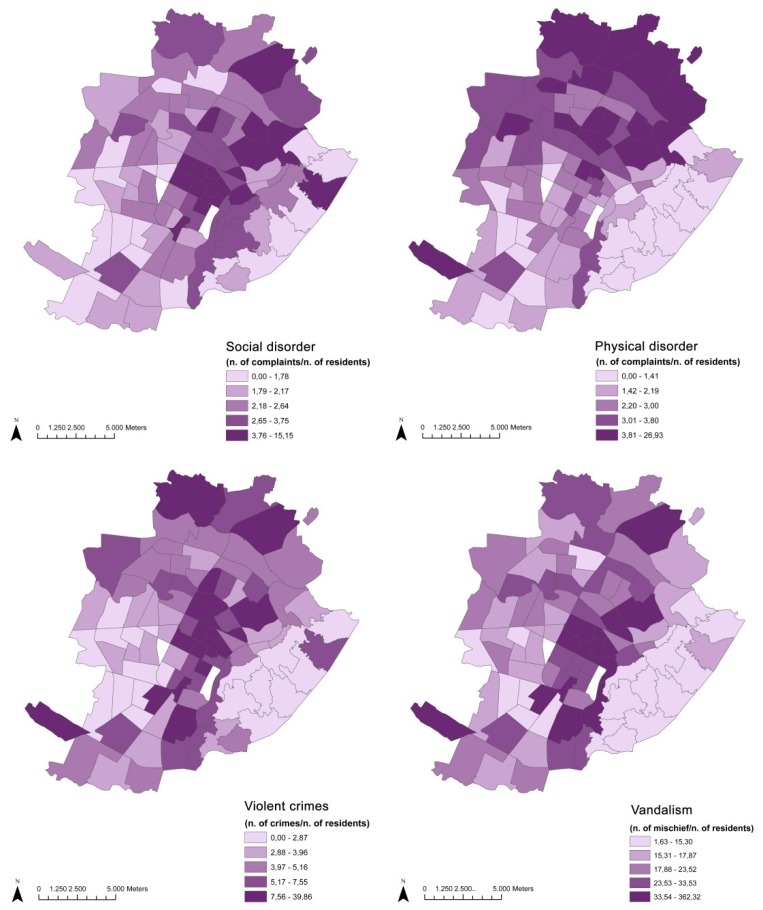
Distribution of SE variables.

Neighborhood SE variables refer to the occurrence among the resident population of urban safety and neighborhood perception, as measured through municipal administrative registries (Figure 4). Social disorder refers to particular complaints made to the municipal police for noise, conflicts and annoyance, public disturbance, improper use of public space and disturbing behaviour in general. This phenomenon can have a marked influence on people’s perception of safety and micro-conflicts, and disturbances can provoke or inflame existing friction in the urban context. There is evidence of an increasing trend in recent years which may be due to a lowering of personal tolerance thresholds or to changing behaviours in the use of public spaces, which are more diversified and are taking place throughout both the day and night times.

Physical disorder refers to complaints made to municipal authorities for squalor, urban decay and acts of vandalism to garbage bins. This indicates a general carelessness in the area and could affect the sense of safety felt by residents due to the perception that the area is abandoned or of little interest to public authorities.

Violent crimes refer to crimes involving physical aggression, such as homicide (all typologies), fights, insults, assaults and battery, kidnapping and sexual violence (and related offenses). It is worth considering that most of these are reported to the police because they occurred in public (therefore these do not cover all crimes that are unreported and taking place in private).

Vandalism refers to criminal offences committed against public goods that are reported to or discovered by the police. It includes criminal offences commonly falling under the definition of vandalism. It is worth noting that these offences may have varied definitions within the diverse legal systems of European countries.

Each of these area-based variables was dichotomized using the median split procedure. This choice was suggested by the observed distribution, as linearity of covariates was not always respected, and it meets the need of simplification, as we could only estimate one parameter per variable due to model complexity. The literature did not offer useable solid cut-off values, consequently we opted for the median split. The values are reported in Table 1.

**Table 1:** Cut-off values.

Covariate	Median (Cut-Off Value)	Min Value	Max Value
Functional mix	56	1	100
Accessibility by public transport	32	23	49
Cultural leisure and sport facilities	59.63	7.45	100
Green and pietonal areas	42.30	2.53	78.62
Urban density	1.35	0.07	3.96
Vandalism	22.01	12.29	362.32
Violent crimes	4.79	1.88	39.86
Physical disorder	2.44	1.10	15.15
Social disorder	2.82	0.99	22.43

With regards to the health outcome, the adopted case definition was “at least one antidepressant prescription from the National Health Services during the observation period” (2004–2006). The antidepressants were identified in the DPR by their Anatomical Therapeutic Chemical (ATC) classification code (N06A) (WHO Collaborating Centre for Drug Statistics Methodology 2004) and included: monoamino-oxidase inhibitors (MAOI), selective serotonin uptake inhibitors (SSRI), tricyclic antidepressants (TCA) and others.

Out-of-pocket purchases of drugs were not included in the database and are therefore excluded from the analyses.

The outcome was tested as a function of individual socioeconomic variables (educational level, citizenship, activity status, residential stability), contextual BE variables (density, functional mix, green and pedestrian areas, cultural and leisure facilities, accessibility by public transport) and contextual SE variables (social disorders, physical disorders, violent crimes, vandalism), in order to identify the BE dimensions affecting MH, irrespective of individual and contextual SE disadvantage. We used Poisson multilevel regression models (treating first prescriptions as single events, as in a mortality analysis) stratified by gender and age groups (internally age-standardized), with individuals nested within neighborhoods, to estimate Incidence Rate Ratios (IRR). We introduced in the models the individual socio-demographic set of variables and added one by one the BE predictors as variables of interest (Models A report results of separate regressions with just one BE covariate at a time); we selected the BE significant variables from Model A—and inserted them in Model B, where we added the SE indicators as control variables, leaving in the final model only the significant BE and SE predictors (Models B).

Finally, in order to better highlight any potential social inequalities in vulnerability to the BE effect, we tested the interaction between the most significant variables from the individual socioeconomic set and from the BE set.

Statistical analyses were performed using the STATA 10 software (StataCorp LP, College Station, TX, USA)

## 3. Results and Discussion

### 3.1. Results

Table 2 reports the distribution (n and %) of the whole study population (aged 20–64) and the percentage of individuals who had any antidepressant prescription. As expected, prescription increases with female gender, age, low social status (low education and not being active, with a strong underuse by migrants).

**Table 2:** Study population and first drug prescriptions (2004–2006) by individual characteristics. Turin, age 20–64.

Variable	*N*	*N*	First Prescriptions	% First Prescriptions
*Gender*				
male	272,516	49.80%	16,691	6.12%
female	274,747	50.20%	34,010	12.38%
TOT	547,263		50,701	9.26%
*Age*				
20–34	168,979	30.88%	9029	5.34%
35–49	204,802	37.42%	18,996	9.27%
50–64	173,482	31.70%	22,676	13.07%
*Educational level*				
high	276,598	50.54%	23,460	8.48%
low	270,048	49.35%	27,238	10.09%
missing	617	0.11%	3	0.50%
*Citizenship*				
Italian	496,283	90.68%	49,288	9.93%
foreign	50,411	9.21%	1413	2.80%
missing	569	0.11%		
*Activity status*				
active	286,517	52.35%	25,341	8.84%
non active	162,808	29.75%	19,435	11.94%
missing	97,938	17.9%	5925	6.05%
*Residential stability*				
resident	463,073	84.62%	43,492	9.39%
not resident	71,737	13.11%	5873	8.19%
missing	12,453	2.27%	1336	10.73%

In Table 3, we present results of the multivariable models A and B. Model A accounts for individual socio-demographic set of variables and BE covariates added one by one; Model B presents results for individual socio-demographic set of variables plus significant BE and SE covariates.

**Table 3:** Prescriptions of antidepressant (2004–2006) according to individual and neighborhood characteristics. Incidence Rate Ratios (IRR) by age and gender. Turin, 20–64 years.

**MEN (IRR)**							
**Model ^1^**	**20–34**		**35–49**		**50–64**		
	**A**	**B**	**A**	**B**	**A**	**B**	
*Educational level* (*low*)	1.16 (1.07; 1.26)	1.16 (1.07;1.26)	0.98 (0.93; 1.04)	0.98 (0.93; 1.04)	**0.85 (0.81; 0.90)**	**0.86 (0.82; 0.91)**	
*Citizenship* (*foreign*)	**0.29 (0.21; 0.41)**	**0.29 (0.21;0.41)**	**0.25 (0.20; 0.32)**	**0.25 (0.20; 0.32)**	**0.31 (0.20; 0.47)**	**0.31 (0.20; 0.47)**	
*Residential stability* (*<3 years. at address*)	1.01 (0.91; 1.12)	1.01 (0.91;1.12)	**0.9 (0.83; 0.97)**	**0.9 (0.83; 0.97)**	**0.69 (0.63; 0.75)**	**0.69 (0.63; 0.75)**	
*Activity status* (*non active*)	**1.49 (1.37; 1.64)**	**1.49 (1.37;1.64)**	**1.92 (1.78; 2.06)**	**1.92 (1.78; 2.06)**	1.34 (1.27; 1.42)	1.34 (1.27; 1.42)	
*Urban density* (*high*)	0.99 (0.91; 1.09)		0.98 (0.93; 1.04)		**0.92 (0.86; 0.97)**		
*Public transport* (*high accessibility*)	0.95 (0.87; 1.04)		0.99 (0.94; 1.05)		**0.92 (0.87; 0.97)**	**0.93 (0.87; 0.98)**	
*Green and pietonal areas* (*high*)	1.03 (0.94; 1.13)		0.99 (0.93; 1.05)		0.98 (0.92; 1.04)		
*Cultural leisure and sport facilities* (*high*)	1.07 (0.99; 1.17)		0.98 (0.93; 1.04)		0.97 (0.91; 1.03)		
*Functional mix* (*high*)	1.02 (0.94; 1.12)		1.00 (0.94; 1.06)		0.99 (0.93; 1.05)		
*Physical disorder* (*high*)						1.07 (1.01; 1.14)	
**WOMEN (IRR)**							
**Model**	**20–34**		**35–49**		**50–64**		
	**A**	**B**	**A**	**B**	**A**	**B.1**	**B.2**
*Educational level (low)*	**1.2 (1.14;1.29)**	**1.2 (1.14;1.29)**	**1.12 (1.08;1.17)**	**1.12 (1.08;1.17)**	**0.94 (0.91;0.98)**	**0.94 (0.91;0.98)**	**0.94 (0.91;0.98)**
*Citizenship (foreign)*	**0.4 (0.32;0.48)**	**0.4 (0.32;0.48)**	**0.49 (0.42;0.56)**	**0.49 (0.42;0.56)**	**0.42 (0.33;0.55)**	**0.42 (0.33;0.55)**	**0.42 (0.33;0.55)**
*Residential stability (<3 yrs at address)*	0.98 (0.90;1.06)	0.98 (0.90;1.06)	**0.9 (0.84;0.96)**	**0.9 (0.84;0.96)**	**0.77 (0.71;0.82)**	**0.77 (0.71;0.82)**	**0.77 (0.71;0.82)**
*Activity status (non active)*	**1.14 (1.07;1.22)**	**1.14 (1.07;1.22)**	**1.15 (1.11;1.20)**	**1.15 (1.11;1.20)**	**1.19 (1.15;1.23)**	**1.19 (1.15;1.23)**	**1.19 (1.15;1.23)**
*Urban density (high)*	0.96 (0.90;1.02)		0.98 (0.94;1.02)		**0.95 (0.92;0.98)**		**0.96 (0.92;0.99)**
*Public transport (high accessibility)*	**0.94 (0.88;0.99)**	**0.94 (0.88;0.99)**	**0.95 (0.92;0.99)**	**0.95 (0.92;0.99)**	**0.95 (0.92;0.98)**	**0.95 (0.92;0.98)**	
*Green and pietonal areas (high)*	0.97 (0.90;1.02)		0.96 (0.91;1.01)		1.00 (0.96;1.08)		
*Cultural leisure and sport facilities (high)*	0.97 (0.91;1.03)		0.97 (0.93;1.01)		0.99 (0.96;1.03)		
*Functional mix (high)*	0.94 (0.89;1.00)		0.98 (0.94;1.02)		1.00 (0.96;1.03)		
*Physical disorder (high)*						**1.04 (1.01;1.08)**	**1.04 (1.01;1.08)**

^1^ Models A: xi: (Individual variables) + BE added singularly. Models B: xi: (Individual variables) + BE + SE (final model with significant estimates).

In Models A, each BE indicator was adjusted for all individual socioeconomic indicators. Looking at the main individual predictors of prescriptions for antidepressants, employment status shows a stronger effect for men, with a peak for those aged between 35 and 49 years. Values for women are still significant, but the effect is weaker and comparable across ages. Low school attainment is associated with higher risk among younger men (20–34) and women (20–49), while it is a slightly protective factor for elderly individuals of both sexes. Immigrant status is associated with a consistently lower use of drugs in both sexes and all age groups, although the rates are somewhat higher among men. Residential stability is associated with a lower incidence of drug prescription for both sexes aged 35–65 years, which increases with age. Residential stability does not seem to have an impact on youth between the ages of 20 and 34 years.

Among BE indicators for men, accessibility by public transport and urban density seem to influence the variations of antidepressant prescriptions only after age 50. For women, the protective impact of accessibility by public transport is statistically significant in each age group, and urban density only has a protective impact after the age of 50.

The final Models B include the statistically significant BE and SE neighborhood variables adjusted for all individual characteristics. Due to their high collinearity (0.67), the specific effects of urban density and accessibility by public transport were nullified when jointly inserted in the model. As a result, we decided to undertake parallel analyses (models B.1 and B.2). The results are comparable for both indicators, demonstrating a significant protective effect of the BE covariate only for the elderly, accompanied by a statistically significant risk that is attributable to physical disorder.

The interaction between employment status and accessibility by public transport (the individual and neighborhood variables with the most significant impact on antidepressant prescription) was tested and found to have insignificant results.

### 3.2. Discussion

In Turin, the incidence of depressive symptoms among adults, as measured by any prescription of antidepressant drugs, decreases with the improvement of some specific features of the urban BE. This is primarily related to the protective effect of *urban density* and of *accessibility by public transport*, with stronger effects evident among women and older adults. This association is true irrespective of the individual level of social disadvantage and the degree of challenges in the neighborhood.

The association between social inequalities and the manifestation of depressive symptoms is well documented in epidemiological literature [34,35,36,37]. The strongest association has been observed in relation to unemployment, lower education, low income and some material deprivation and most severely affects women of low SES [26,38,39],children, adolescents [40,41] and the elderly [42].

In our data, a low level of education and inactivity shows the strongest effect on the incidence of first antidepressant prescriptions, particularly among unemployed men.

SE covariates do not show any effect in our study population when adjusted by individual social position, apart from a small increase of risk evidenced among the elderly related to exposure to physical disorder. Physical disorder refers to a perception of urban decay as measured by complaints to local authorities and is strongly related to a sense of safety. This has been identified as one of the key determinants of health outcomes, particularly MH, in the literature [43]. Since information about the SE is drawn from reported complaints, we cannot exclude the possibility of a bias that is due to over notification or misclassification.

No previous study has specifically investigated the contribution of individual features of the BE, however, there has been general reporting that the BE has an effect on MH, both through direct and indirect pathways. Many studies have tried to shed a light on those pathways, but this has been primarily done by focusing on one determinant at a time (green infrastructures or accessibility to basic services, *etc.*) without considering the complexity of the general urban structure.

Direct pathways have been primarily identified through physical indoor housing and working conditions, and the effects on health are well known and documented in the literature [8,44,45].

Home and work environments are the BE determinants with strongest health effects, as those are the places where people spend the majority of their time. The direct effects on health due to the outdoor urban environment is more difficult to isolate and is recognized to be of minor intensity.

Moreover, the urban BE can have an important impact on MH through indirect pathways. The literature has primarily paid attention to two pathways that are somehow related to the SE, namely social capital/networks and support, which we were unable to measure in our study, and urban safety and perception of neighborhood disorder, which we have measured through SE variables. In order to isolate the independent effects of the BE, we used the SE variables as control variables in the complete model. Many studies have identified fear of crime as one of the determinants contributing to negative MH in urban contexts [19,46], as it can lead to suppressed activities and unfulfilled needs, as well as reduced social interactions. In our research, *physical disorder* approximates these results.

What is evident from our model is that accessibility by public transport and urban density are the two principal features of the BE that contribute to some antidepressant drug use among the study population. We relied on the literature for selecting our BE variables of interest, particularly as there are a large number of studies focused on the connections between MH and green spaces (findings recently summarized in the review by Lee *et al.* [47]), mixed land use [45,48], or that highlight the importance of having good proximity to public services [44]. In our results, however, these connections were not evident.

On the one hand, this is coherent with the latest research findings. Lee *et al.* [47] found that while many studies tried to assess the evidence linking urban green spaces to positive health benefits, the results are “weak, inconsistent, and occasionally contradictory” ([47], p. 134). This was hypothesized to be due to the heterogeneity of the studies undertaken, as well as to limitations affecting observational, ecological and cross-sectional studies, notably multiple confounding factors and the long time lag between exposure to urban structure and the manifestation of effects.

On the other hand, and more specifically in connection with our study design, this absence of significant associations may be due to the fact that the effect of some urban features has been hidden by and included in the notions of density and accessibility. These are in turn mutually exclusive when inserted into the model. In other words, urban density—at least in European cities and particularly in Turin—is associated with a variety of uses, urban vitality and a pleasant BE. This primarily coincides with city centers which are often characterized by high accessibility due to the availability of reliable connections and easy access to any services.

In our results, the younger population—especially men—seem to be less reactive to their neighborhood environments, both social and structural. On average, women and the elderly tend to pass longer periods of time at home. Some scholars [49,50] suggest the effect of the neighborhood (its BE and SE components) are dose-related. Our research has confirmed that women, the elderly and those who have been residentially stable are more affected by their neighborhood environments. Fewer prescriptions have been given to women and elderly individuals living in areas well-serviced by public transport. Women, who on average live closer to their workplaces, use more public transport (higher number of trips per day) and walk more in comparison to men [51,52,53]. Scholars concerned with equality issues have highlighted the differences in the activities conducted by the two genders during the day. According to Hurez and Richer, “the location of men and women in time and space is a reflection of a territory’s characteristics” [54], closely linked to the nature and location of economic activities, but also to the availability of facilities and services.

In fact, the transportation system enables individuals to access a wider variety of activities. Even if services cannot be distributed within the close proximity of every resident, public transport helps to close the gap by giving individuals the opportunity to easily and quickly reach the needed service. Car use increases as we move further outside the city center toward the suburbs [55]. Public transport is not as developed as in the city center and living in a suburban area without a car can have a serious impact on MH, particularly when it results in forgone trips, especially for older adults.

From the point of view of health inequalities, the protective effect of accessibility is evenly distributed across the population by educational level and employment status. This means that these two coefficients are independent determinants of health inequalities in the population.

### 3.3. Limits of the Study

The MH could have been better measured by means of psychometric questionnaires, but it would have reduced the analysis to a sample. The TLS, however, by relying on census and administrative data, aims at surveying the whole population and therefore provides a stronger statistical power.

Only prescriptions issued through the National Health System were considered in the study and out-of-pocket purchases were excluded. It is recognized that this could lead to an underestimation of consumption by those who are economically privileged, as we may suppose that this group can more easily afford private medical expenses. In addition, a bias could also be identified in the issuance of NHS prescriptions as antidepressants are surely an inconclusive indicator of the full extent of depressive symptoms, given that seeking care (or not) is frequently influenced by various social and cultural factors, while receiving a prescription for an antidepressant will vary greatly in accordance with physicians’ attitudes. Depression is severely underdiagnosed and undertreated, even in developed countries [56,57]. Therefore, we can infer that cases identified through antidepressants only represent a subset of those with depressive symptoms and that subjects with milder symptoms are likely underrepresented in our case series.

We have previously mentioned some omitted variables in the control of confounding factors, More specifically, better measures of social disadvantage both at the individual level (*i.e.*, income) and at the area level (*i.e.*, social capital) could have improved the validity of our estimates. The small protective effect of some BE variables, however, appears so strongly independent of individual and area social conditions that a better measure is unlikely to change the direction of the association. Some other important aspects of the physical urban health context are missing, which could have helped explain indirect pathways, such as environmental stressors (pollution, noise, air quality, *etc.*); however, reliable up-to-date data on air quality and noise pollution was unavailable at the neighborhood level.

In addition, the scope of analysis may not have been specific enough to show the health effects of particular BE factors, such as green spaces. Some scholars [49] suggest studies that focus on smaller areas, depending on the pathways (determinant-outcome association) that are under study. Although we know that statistical zones in Turin are quite homogeneous, taking micro-level BE data (such as census tracts) into account in the analyses could enrich and expand our results.

## 4. Conclusions

This study explores the contribution of specific features of the urban structure and distribution of services on minor depressive disorders in the city of Turin, controlling for individual socioeconomic factors and SE variables. The large size of the population and its statistical power, the cohort design, the ability to control residential mobility and the main social determinants are the main strengths of the study.

In conclusion, the results of this research suggest that the distribution of prescriptions for antidepressants is, in addition to known factors, slightly influenced by some components of the BE (accessibility by public transport and urban density), particularly among the elderly and women. The BE seems unable to modify the effect of some socioeconomic characteristics, that is to say, to influence the capacity and resilience of the most vulnerable to confront social and economic distress.

In order to address health inequalities, urban policies should invest in the delivery of services that enhance resilience factors, above all a good public transport network, in a careful and equal manner, throughout the city. Attention must be paid to more vulnerable groups, such as the elderly and particularly women. Accessibility and transport services could be easily modified by policymakers as a compensation measure when it is impossible to provide proximity to services for everyone.

Such efforts could contribute to the improvement of MH in the city in relation to urban policies. It should also be remembered that the main responsibility for the promotion of MH remains with the social policy sector, to foster equity in the distribution of social determinants.

## References

[1] Ståhl T., ismar M., Lila E., Lahtine E., Leppo K. (2006). Health in all Policies: Prospects and Potentials.

[2] Dalgard O.S., Tambs K. (1997). Urban environment and mental health. A longitudinal study. Br. J. Psychiatry.

[3] Ross C.E. (2000). Neighborhood disadvantage and adult depression. J. Health Soc. Behav..

[4] Duncan C., Jones K., Moon G. (1995). Psychiatric morbidity: A multilevel approach to regional variations in the UK. J. Epidemiol. Community Health.

[5] Pickett K.E., Pearl M. (2001). Multilevel analyses of neighbourhood socioeconomic context and health outcomes: A critical review. J. Epidemiol. Community Health.

[6] Reijneveld S.A., Schene A.H. (1998). Higher prevalence of mental disorders in socioeconomically deprived urban areas in The Netherlands: Community or personal disadvantage?. J. Epidemiol. Community Health.

[7] Surtees P., Wainwright N., Luben R., Khaw K.T., Day N. (2003). Sense of coherence and mortality in men and women in the EPIC-Norfolk United Kingdom prospective cohort study. Am. J. Epidemiol..

[8] Weich S., Blanchard M., Prince M., Burton E., Erens B., Sproston K. (2002). Mental health and the built environment: Cross-sectional survey of individual and contextual risk factors for depression. Br. J. Psychiatry.

[9] Weich S., Holt G., Twigg L., Jones K., Lewis G. (2003). Geographic variation in the prevalence of common mental disorders in Britain: A multilevel investigation. Am. J. Epidemiol..

[10] Araya R., Dunstan F., Playle R., Thomas H., Palmer S., Lewis G. (2006). Perceptions of social capital and the built environment and mental health. Soc. Sci. Med..

[11] Mitchell D. (2003). The Right to the City: Social Justice and the Fight for Public Space.

[12] Frank L.D., Kavage S. (2008). Urban planning and public health: A story of separation and reconnection. J. Public Health Manag. Pract..

[13] Duncan D.T., Piras G., Dunn E.C., Johnson R.M., Melly S.J., Molnar B.E. (2013). The built environment and depressive symptoms among urban youth: A spatial regression study. Spat. Spatiotemporal Epidemiol..

[14] Dreger S., Buck C., Bolte G. (2014). Material, psychosocial and sociodemographic determinants are associated with positive mental health in Europe: A cross-sectional study. BMJ Open.

[15] Guite H., Clark C., Ackrill G. (2006). The impact of the physical and urban environment on mental well-being. Public Health.

[16] Annerstedt M., Ostergren P.O., Björk J., Grahn P., Skärbäck E., Währborg P. (2012). Green qualities in the neighbourhood and mental health—Results from a longitudinal cohort study in Southern Sweden. BMC Public Health.

[17] Kirkbride J.B., Fearon P., Morgan C., Dazzan P., Morgan K., Murray R.M., Jones P.B. (2007). Neighbourhood variation in the incidence of psychotic disorders in Southeast London. Soc. Psychiatry Psychiatr. Epidemiol..

[18] Fone D., Dunstan F., Lloyd K., Williams G., Watkins J., Palmer S. (2007). Does social cohesion modify the association between area income deprivation and mental health? A multilevel analysis. Int. J. Epidemiol..

[19] Curry A., Latkin C., Davey-Rothwell M. (2008). Pathways to depression: The impact of neighborhood violent crime on inner-city residents in Baltimore, Maryland, USA. Soc. Sci. Med..

[20] Ellaway A., Morris G., Curtice J., Robertson C., Allardice G., Robertson R. (2009). Associations between health and different types of environmental incivility: A Scotland-wide study. Public Health.

[21] Dannon P.N., Lowengrub K., Iancu I., Kotler M. (2004). Paroxetine in panic disorder: Clinical management and long-term follow-up. Expert Rev. Neurother..

[22] Hansen R.A., Gaynes B.N., Gartlehner G., Moore C.G., Tiwari R., Lohr K.N. (2008). Efficacy and tolerability of second-generation antidepressants in social anxiety disorder. Int. Clin. Psychopharmacol..

[23] D’Errico A., Cardano M., Landriscina T., Marinacci C., Pasian S., Petrelli A., Costa G. (2011). Workplace stress and prescription of antidepressant medications: A prospective study on a sample of Italian workers. Int. Arch. Occup. Environ. Health.

[24] Kivimäki M., Gunnell D., Lawlor D.A., Smith G.D., Pentti J., Virtanen M., Elovainio M., Klaukka T., Vahtera J. (2007). Social inequalities in antidepressant treatment and mortality: A longitudinal register study. Psychol. Med..

[25] Bocquier A., Cortaredona S., Verdoux H., Sciortino V., Nauleau S., Verger P. (2013). Social inequalities in new antidepressant treatment: A study at the individual and neighborhood levels. Ann. Epidemiol..

[26] Hansen D.G., Søndergaard J., Vach W., Gram L.F., Rosholm J.U., Mortensen P.B., Kragstrup J. (2004). Socio-economic inequalities in first-time use of antidepressants: A population-based study. Eur. J. Clin. Pharmacol..

[27] Hollander A.C. (2013). Social inequalities in mental health and mortality among refugees and other immigrants to Sweden—Epidemiological studies of register data. Glob. Health Action.

[28] Brendler-Lindqvist M., Norredam M., Hjern A. (2014). Duration of residence and psychotropic drug use in recently settled refugees in Sweden-a register-based study. Int. J. Equity Health.

[29] Marinacci C., Spadea T., Biggeri A., Demaria M., Caiazzo A., Costa G. (2004). The role of individual and contextual socioeconomic circumstances on mortality: Analysis of time variations in a city of north west Italy. J. Epidemiol. Community Health.

[30] Stringhini S., Spadea T., Stroscia M., Onorati R., Demaria M., Zengarini N., Costa G. (2014). Decreasing educational differences in mortality over 40-years: Evidence from the Turin Longitudinal Study (Italy). Eur. J. Public Health.

[31] Melis G., Masala E., Tabasso M. (2015). From the smart city to the people-friendly city: Usability of tools and data. Handbook of Research on Social, Economic, and Environmental Sustainability in the Development of Smart Cities.

[32] Shannon C.E. (1948). A mathematical theory of distribution. Bell Syst. Techn..

[33] Frank L.D., Pivo G. (1994). Impacts of Mixed Use and Density on Utilization of Three Modes of Travel: Single-Occupant Vehicle, Transit, and Walking.

[34] Fryers T., Melzer D., Jenkins R. (2003). Social inequalities and the common mental disorders. Soc. Psychiatry Psychiatr. Epidemiol..

[35] Fryers T., Melzer D., Jenkins R., Brugha T. (2005). The distribution of the common mental disorders: Social inequalities in Europe. Clin. Pract. Epidemiol. Ment. Health.

[36] Lorant V., Deliège D., Eaton W., Robert A., Philippot P., Ansseau M. (2003). Socioeconomic inequalities in depression: A meta-analysis. Am. J. Epidemiol..

[37] Sundquist K., Ahlen H. (2006). Neighbourhood income and mental health: A multilevel follow-up study of psychiatric hospital admissions among 4.5 million women and men. Health Place.

[38] Green M.J., Benzeval M. (2013). The development of socioeconomic inequalities in anxiety and depression symptoms over the lifecourse. Soc. Psychiatry Psychiatr. Epidemiol..

[39] Green M.J., Espie C.A., Benzeval M. (2014). Social class and gender patterning of insomnia symptoms and psychiatric distress: A 20-year prospective cohort study. BMC Psychiatry.

[40] Klanšček H.J., Ziberna J., Korošec A., Zurc J., Albreht T. (2014). Mental health inequalities in Slovenian 15-year-old adolescents explained by personal social position and family socioeconomic status. Int. J. Equity Health.

[41] Reiss F. (2013). Socioeconomic inequalities and mental health problems in children and adolescents: A systematic review. Soc. Sci. Med..

[42] Saarloos D., Alfonso H., Giles-Corti B., Middleton N., Almeida O.P. (2011). The built environment and depression in later life: The health in men study. Am. J. Geriatric Psychiatry.

[43] Almedom A.M. (2005). Social capital and mental health: An interdisciplinary review of primary evidence. Soc. Sci. Med..

[44] Evans G.W. (2003). The built environment and mental health. J. Urban Health.

[45] Renalds A., Smith T.H., Hale P.J. (2010). A systematic review of built environment and health. Fam. Community Health.

[46] Latkin C.A., Curry A.D. (2003). Stressful neighborhoods and depression: A prospective study of the impact of neighborhood disorder. J. Health Soc. Behav..

[47] Lee A., Maheswaran R. (2011). The health benefits of urban green spaces: A review of the evidence. J. Public Health.

[48] Sturm R., Cohen D.A. (2004). Suburban sprawl and physical and mental health. Public Health.

[49] Galster G. (2008). Quantifying the effect of neighbourhood on individuals: Challenges, alternative approaches, and promising directions. Schmollers Jahrb..

[50] Vallée J., Cadot E., Roustit C., Parizot I., Chauvin P. (2011). The role of daily mobility in mental health inequalities: The interactive influence of activity space and neighbourhood of residence on depression. Soc. Sci. Med..

[51] Roux S. (2012). Transition of motorisation in France in the 20th century. Ph.D Thesis.

[52] Knoll B., Szalai E. (2005). Gender Mainstreaming und Mobilität in Niederösterreich.

[53] McDonald N.C. (2005). Does residential density affect the travel “gender gap”. Conference Proceedings 35: Research on Women’s Issues in Transportation.

[54] Hurez C., Richer C. Does the City’s Pulse Beat at the SAME RAte for Men and Women? Gender Time-Geography. Proceedings of the Women’s Issues in Transportation.

[55] Rosenbloom S. (2006). Understanding women’s and men’s travel patterns. Research on Women’s Issues in Transportation: Report of a Conference.

[56] Davidson J.R., Meltzer-Brody S.E. (1999). The underrecognition and undertreatment of depression: What is the breadth and depth of the problem?. J. Clin. Psychiatry.

[57] Demyttenaere K., Bruffaerts R., Posada-Villa J., Gasquet I., Kovess V., Lepine J.P., Angermeyer M.C., Bernert S., de Girolamo G., Morosini P. (2004). Prevalence, severity, and unmet need for treatment of mental disorders in the World Health Organization World Mental Health Surveys. JAMA.

